# *Agrobacterium*-Mediated Transformation of the Dwarf Soybean MiniMax

**DOI:** 10.3390/plants13071013

**Published:** 2024-04-02

**Authors:** Min Shao, Kent F. McCue, James G. Thomson

**Affiliations:** USDA-ARS Crop Improvement and Genetics, Western Regional Research Center, Albany, CA 94710, USA; min.shao@usda.gov (M.S.); kent.mccue@usda.gov (K.F.M.)

**Keywords:** soybean, MiniMax cultivar, transformation, regeneration, phloroglucinol, mycorrhiza fungi

## Abstract

This study aims to establish an *Agrobacterium*-mediated transformation system for use with the ‘MiniMax’soybean cultivar. MiniMax is a mutant soybean whose growth cycle is around 90 days, half that of most other soybean varieties, making it an optimal model cultivar to test genes of interest before investing in modification of elite lines. We describe an efficient protocol for *Agrobacterium*-mediated transformation using MiniMax seeds. It uses a modified ‘half seed’ regeneration protocol for transgenic soybean production, utilizing the rapid generation MiniMax variety to obtain T1 seeds in approximately 145 days. Addition of phloroglucinol (PG) to the regeneration protocol was key to obtaining high-efficiency rooting of the regenerated shoots. Transfer to soil was accomplished using an organic soil amendment containing nutrients and mycorrhiza for plants to thrive in the greenhouse. This combination of genotype and stimulants provides a transformation protocol to genetically engineer MiniMax seeds with a transgenic lab-to-greenhouse production efficiency of 4.0%. This is the first report of MiniMax soybean whole plant transformation and heritable T1 transmission. This protocol provides an ideal resource for enhancing the genetic transformation of any soybean cultivar.

## 1. Introduction

Soybean (*Glycine max* L. Merrill) is a globally significant source of nutrition, both as whole seed and when processed for protein and oil. Improving nutritional content, disease resistance, and environmental tolerance requires the ability to efficiently introduce modified and novel genes conferring new traits and phenotypes. Many advanced biotechnology techniques require the ability to genetically alter the genome and regenerate whole plants from callus or tissue culture. Further analysis and segregation of desirable traits also requires the ability to rapidly produce seeds for future generational analysis. Regeneration of soybean subsequent to *Agrobacterium* transformation has been historically difficult and time consuming. In addition, the typical generation time of soybean (~270 days) makes it difficult to rapidly obtain seeds for analysis.

**Figure 1:**
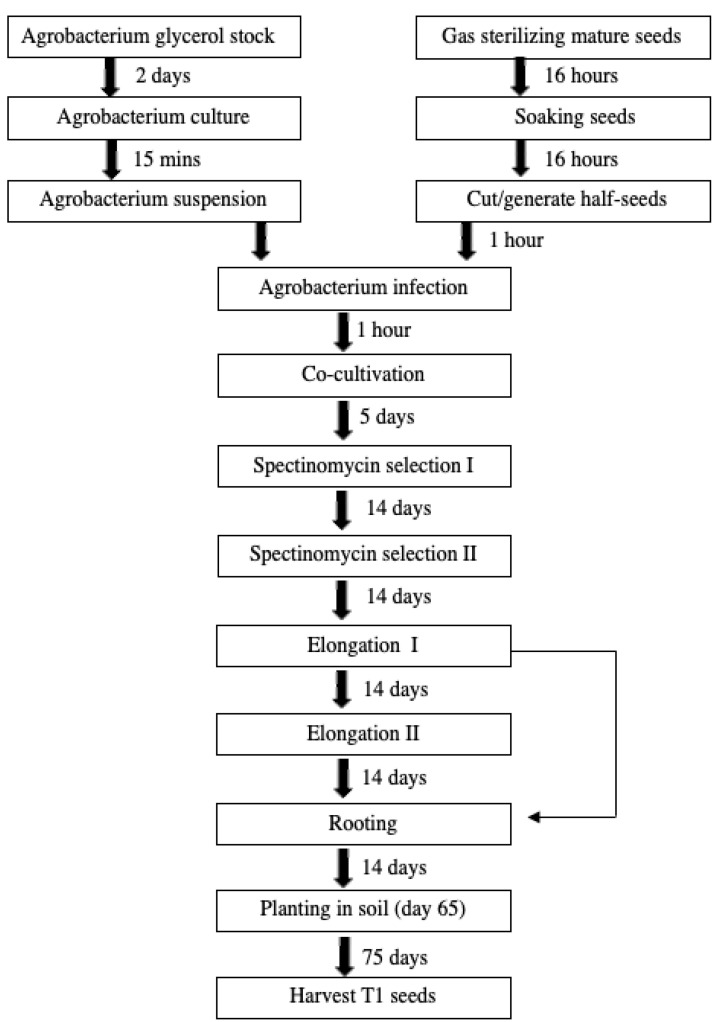
Flow chart of *Agrobacterium*-mediated transformation using Minimax mature half-seed transformation through to harvest of T1 seeds. Approximately 145-day procedure.

**Figure 2:**
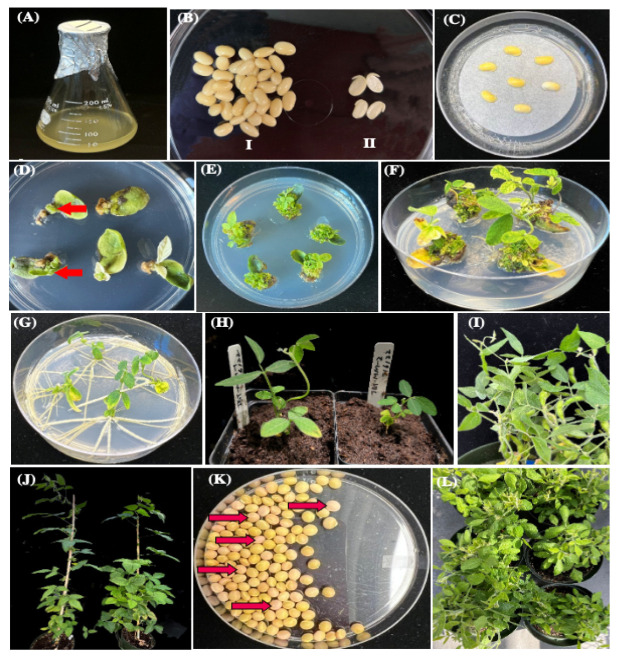
Agrobacterium-mediated soybean transformation workflow. The workflow protocol requires up to nine weeks from seeds to transgenic seedlings (**A**–**G**). (**A**) *Agrobacterial* culture, (**B**) (I) Sterile seeds after hydration, (II) seed in ‘half-seed’ configuration ready for transformation). (**C**) Half-seed co-cultivation with filter paper, (**D**) Spectinomycin resistant tissues after two weeks (green (red arrows) vs. white shoots), (**E**) Spectinomycin-resistant tissues on elongation medium, (**F**) Spectinomycin-resistant shoots ready for transfer to RM, (**G**) Rooted transgenic shoots on RM, (**H**) Rooted transgenic shoots growing in soil, (**I**) Transgenic soybean plants in the greenhouse. (**J**) Putative transgenic T0 plants vegetative stage. (**K**) Identification of T1 mCherry3 positive seeds (pink seeds-red arrows point out examples). (**L**) T1 transgenic plants in reproductive stage.

MiniMax soybean [*Glycine max* (L.) Merr.] is a variety developed by the USDA that exhibits compact growth (22 cm) and early maturity (73–85 days), allowing cultivation of more plants in a shorter time in a smaller space [1]. This variety is an ideal model for testing gene delivery and stacking techniques for the ultimate production of improved elite soybean varieties. The process typically requires ~270 days and has a success rate of 2–10%, depending on cultivar and technique employed [2,3,4,5]. Here we report the development of a transformation protocol involving organogenic regeneration in the MiniMax variety that requires ~145 days for T1 seed generation (Figure 1). For plant transformation we utilized the GA*A*NTRY system [6] to facilitate introduction of a selectable marker, a visible reporter gene, and an assayable reporter gene on a single T-DNA (Figure 2). The combination of simple transformation and regeneration in an early maturing variety enables accelerated genetic research for soybean improvement.

## 2. Results

**Figure 3:**
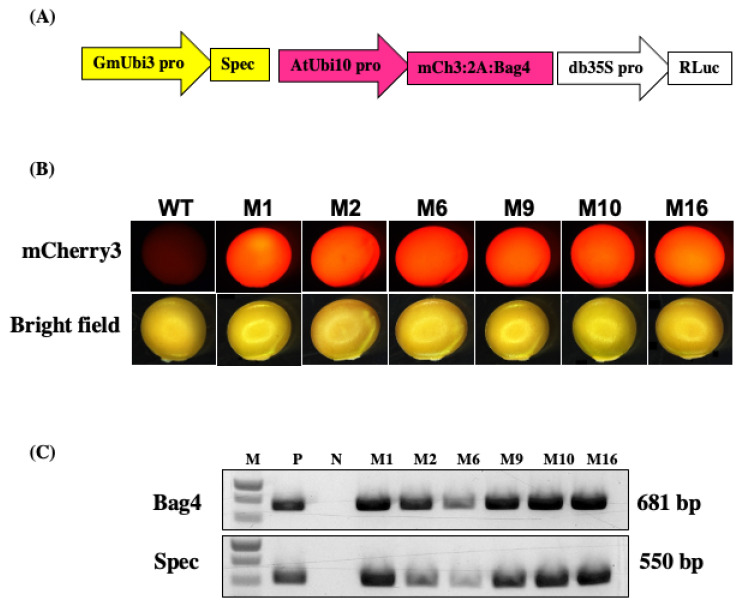
mCherry3 (mCh3) expression in transgenic soybean seeds. (**A**) Schematic diagram of the JGT44 T-DNA (Spec: spectinomycin resistance; mCh3: mCherry3; Rluc: *Renilla* Luciferase; Bag4: Arabidopsis derived Bcl-2-associated athanogene 4; GmUbi3, AtUbi10, and db35S: promoters), (**B**) Representative mCherry3 fluorescence images of dry mature seeds of wild type (WT) and JGT44 transgenic lines (M1, M2, M6, M9, M10 and M16), (**C**) PCR analysis of genomic DNA of putative transgenic soybean plants using BAG4 gene and Spec gene primers. The length of PCR productions is 681 bp and 550 bp, respectively. M: DNA ladder; P: JGT44 gDNA; N: Wild type soybean gDNA; M1, M2, M6, M9, M10, and M16 are representative transformed soybean plants.

A MiniMax soybean transformation method was established in this research. The ‘half seed’ method and steps of MiniMax soybean transformation are illustrated (Figure 1 and Figure 2). Sterile soybean seeds were soaked overnight, cut out, and used for transformation (Figure 2B). The *Agrobacterium*-infected half-seeds were moved to co-cultivation medium (CC) with filter paper (Figure 2C) and incubated in a growth chamber at 24 °C, 18/6 photoperiod, for 5 d. The explants then were transferred to SI containing 40 mg/L spectinomycin, and clustered shoots appeared at around 2 weeks (most were yellow/white; Figure 2D). Most of clustered shoots were removed, except for green shoots and tissue (Figure 2D, red arrow). After a second round of selection on fresh SI medium, the explants were transferred onto shoot elongation medium (SE, Figure 2E). Shoots longer than 4 cm (Figure 2F) were cut and inserted into rooting medium (RM). Roots were seen after 7 days in RM (Figure 2G) and were tested for mCherry3 florescent proteins expression under the microscope. Seedlings that were positive for red fluorescence expression were moved to pots with soil Sunshine Mix 1/FOOP supplemental fertilizer (Figure 2H), kept in a 26 °C growth chamber for 1–2 weeks, then transferred to a greenhouse until T1 seed harvest (Figure 2I–L and Figure 3B).

Media used for the transformation procedure and a step-by-step procedure is provided in the Materials and Methods section. The results of MiniMax transformation and positive seedling screening are described in Table 1.

The unmodified ‘half seed’ protocol [7] is sufficient to regenerate shoots with an efficiency average of 30%. However, during the rooting process, 75% of the regenerated plants failed to produce roots, and those that did root failed to thrive in soil. To combat this, a range of phloroglucinol (PG) concentrations (0.1, 0.5, 1.0 and 2.0 mg/L) were tested to improve rooting efficiency based on studies by [8,9]. Significant attrition was overcome when the hormone phloroglucinol (PG) was added at 1 mg/L to the SI, SE, and RIM. At 0.5 mL/L PG or below, little to no increase in rooting was observed. However, when the PG concentration was 2 mg/L, a large root mass rapidly developed, and the shoots began to fail. From the 599 explants transformed in the presence PG, 181 (30.2%) regenerated shoots that appeared greener and more robust than those without this hormone. Use of PG increased the rate of shoots that rooted from 25% without PG to 84% with 1 mg/L PG. Successful transplant of rooted plants from in vitro to soil propagation for production of T1 seed required the addition of additional nutrients to the soil mixture. We hypothesize that the availability of mycorrhiza, nutrients and micronutrients in an organic form was needed to stimulate vigorous growth of the transplanted plants. Without the additional organic nutrients, the plants failed to grow and eventually died. The transfer to greenhouse generated a total of 24 positive T0 transgenic seedlings, which were determined by mCherry3 expression and PCR analysis of leaf tissue (Supplemental Figures S1 and S2). The regeneration, rooting, and mCherry3 expression rates were 30.2%, 25.4% and 23%, respectively. From the 24 T0 positive plantlets that survived transfer to soil 13 did not produce seed, and the plants that did produce seed four failed to transmit the transgene as determined by a lack of mCherry3 expression in the seed. Rates of transgene transmission ranged from 42% to 100%. Therefore, results indicate that greenhouse survival with a transgene heritability rate of transgenic T1 plants was 4.0%. This showed a significant drop in efficiency between the rate of transformation/rooting and the final plant survival.

**Figure 4:**
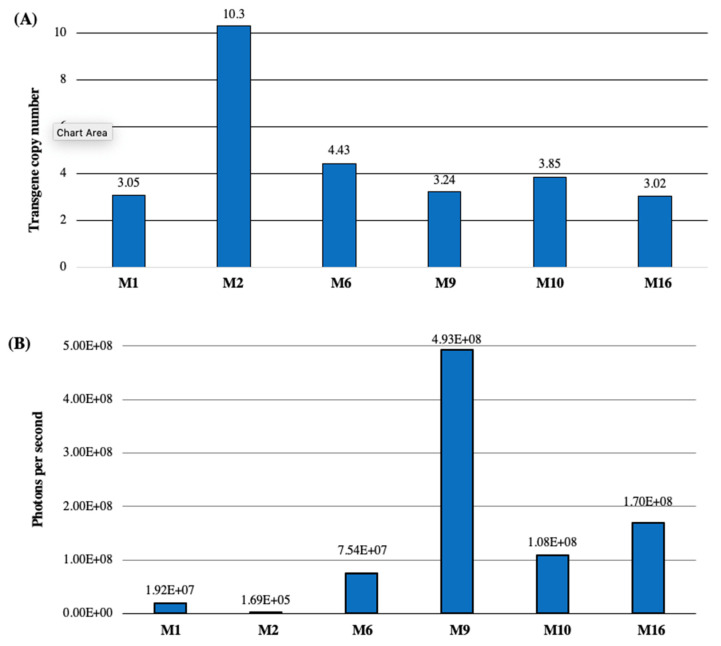
Transgene copy number and Rluc expression. (**A**) Transgene copy number as determined by ddPCR, (**B**) Rluc luminescence for regenerated lines, quantified in photons per second (p/sec). Y-axis: JGT44 transgenic lines (M1, M2, M6, M9, M10 and M16).

Spectinomycin-resistant T0 plants were regenerated from soybean explants inoculated with the GAANTRY line JGT44 (Figure 3A). The mCherry3 reporter gene in the JGT44 construct was used for rapid screening of transformed plants. The mCherry protein was seen as red fluorescence in the developing tissues of spectinomycin-resistant T0 plantlets in vitro (Supplemental Figure S1) and in T1 seeds upon harvest (Figure 3B). PCR positive bands were obtained from red fluorescence leaf tissue of T1 seedlings, confirming germinal transmission from T0 plants (Figure 3C). Tissue from seedlings that were positive for red fluorescence in vitro was harvested to determine T-DNA copy number via ddPCR [10] (Figure 4A). Representative lines displayed T-DNA copy numbers ranging from 3 to 10. Harvested tissue was also used to test Renilla luciferase (Rluc) luminescence, and samples were normalized with a Bradford protein assay. With the exception of line 2, which only displayed background level, remaining plants displayed Rluc luminescence above the background control (Figure 4B).

## 3. Discussion

Using the fast generation MiniMax soybean variety, we have established a method for rapid transformation of soybean to generate transgenic T0 plants. The transformation and regeneration protocol involving organogenic regeneration is a significant improvement over the embryogenic procedure [2]. This method enables high efficiency production of T0 plants, and the MiniMax variety resulted in rapid generation of T1 seeds. Using the GA*A*NTRY system [6] containing the *mCherry3* reporter gene [9] in the transformation improves identification initial transformed lines, as well as rapid selection of transformed seeds. Transformed T1 seeds were obtained at an efficiency of 4.0% in 145 days. While rooting of the derived shoots was initially challenging, addition of PG to the regeneration and rooting media resulted in effective rates of transgenic plant production.

Phloroglucinol (PG) is a phenolic compound that affects a wide range of plant developmental processes [10]. This compound is a precursor in lignin biosynthesis; exogenous application is proposed to play a role in biotic stress resistance, and it has been shown to be beneficial in plant tissue culture [11]. PG increased the number of shoot meristems in potato propagation [12]; increased organogenesis and shoot proliferation in *Ficus* [13]; promoted shoot proliferation and enhanced rooting in *Malus* [14]; and improved rooting efficiency in *Chrysanthemum* [10], *Pongamia* (a legume) [15], citrus [16], and apple [17].

As a component of the lignin biosynthetic pathway, PG is thought to mitigate hyperhydricity by promoting lignification. Hyperhydricity is a common problem in plant propagation affecting revitalization from cryopreservation, transition from liquid culture, and organogenesis and regeneration on semi-solid media. Addition of PG reduces hyperhydricity and increases lignin production in cell cultures of *Vaccinium* [18] and *Achyrocline* [19] and in cultures of *Acca* in immersion bioreactors [20]. Reduction of hyperhydricity can be an important contributor to enhanced rooting efficiency. Addition of PG increased root formation from stem slices of *Malus* [15], rooting of pear rootstock *Pyrus* [21], rooting of micropropagated shoots of *Juglans* [22] and in vitro rooting of *Prunus* [23]. Therefore, PG appears to be an important component for the regeneration and rooting media to improve propagation rates [9].

A final challenge was that plants failed to grow when transferred to a peat/soil mixture (SunShine Mix #1). While healthy, they tended to remain unchanged for weeks before dying—usually of fungus contamination. To stimulate the plants to grow and thrive, an organic soil amendment was added to provide nutrients, minerals, and mycorrhiza in a form that was easy to uptake. In this case, the FOOP (Organic Biosciences, Inc.) soil amendment and leaf spray stimulated the plants to begin growing when transferred to soil for greenhouse growth, going from essentially zero to a 20% rate of survival.

## 4. Materials and Methods

**Experimental Design**

In order to develop a rapid and efficient method for transformation and regeneration of MiniMax soybean we examined the effects of various compounds reported to improve plant transformation. We chose to study the effect of an adjuvant using the ‘half seed’ protocol published by the Wang lab [7]. The study of PG and other modifications to the ‘half seed’ protocol are described below. Table 2 lists media composition used.

**Reagents**

See Supplemental Table S1 for detailed preparation of media.

LB solid medium for *Agrobacterium* plates. Dissolve 10 g tryptone, 5 g yeast extract, 10 g NaCl, and 12.5 g Bacto-agar in water. Bring to a volume of 1 L; pH 7.0 with NaOH; autoclave. Add 50 mL of 100 mg/mL gentamycin stock to 25 mL LB solid medium.

YEB liquid medium for *Agrobacterium* suspension culture. Dissolve 10 g peptone, 5 g yeast extract, and 5 g NaCl in water to make up a total volume of 1 L; pH 7.0 with NaOH. Add 25 mL of 100 mg/mL gentamycin stock to 25 mL YEB liquid medium.

Infection medium (IM). Dissolve 0.32 g Gamborg B5 basal medium, 1 mL Gamborg vitamin (1mg/mL), 30 g sucrose, 3.9 g MES in water to make a total volume of 1 L; pH to 5.4 with KOH. Autoclave for 20 min at 121 °C, 15 psi. After cooling, add GA3 0.25 mg/L, BAP 1.67 mg/L, AS 40 mg/L.

Co-cultivation medium (CCM). Dissolve 0.32 g Gamborg B5 basal medium, 1 mL Gamborg vitamin (1 mg/mL), 30 g sucrose, 3.9 g MES in water to make a total volume of 1 L; pH to 5.4 with KOH. Divide the media into two 1 L bottles (500 mL each). Add 2.125 g of Bacto-agar to each bottle. Autoclave with the lid loose. After cooling, add GA3 0.25 mg/L, BAP 1.67 mg/L, AS 40 mg/L (freshly made), L-Cysteine 400 mg/L, and DTT 154.2 mg/L. Pour into 100 × 15 mm Petri plates. After the medium is solidified, put a piece of sterile filter paper on each plate to reduce bacteria overgrowth.

Shoot induction washing medium (SIW). Dissolve 3.2 g Gamborg B5 medium with vitamins, 30 g sucrose, 0.59 g MES in water to make a total volume of 1 L; pH to 5.7 with KOH. After autoclaving, cool and add BAP 1.67 mg/L, and ticarcillin 200 mg/L.

Shoot induction medium (SI). Dissolve 3.2 g Gamborg B5 medium with vitamins, 30 g sucrose, 0.59 g MES in water to make a total volume of 1 L; pH to 5.7 with KOH. Divide the media into two 1 L bottles, 500 mL each. Add 3.5 g of Bacto-agar to each bottle. Keep the lid loose. After autoclaving, cool and add BAP 1.67 mg/L, PG 1 mg/L, ticarcillin 200 mg/L and spectinomycin 40 mg/L. Pour into sterile 100 × 25 Petri plates.

Shoot elongation medium (SE). Dissolve 4.4 g MS medium with vitamins, 30 g sucrose, 0.59 g MES in water to make a total volume of 1 L; pH to 5.7 with KOH. Divide the media into two 1 L bottles, 500 mL each. Add 3.5 g of Bacto-agar to each bottle. Keep the lid loose. After autoclaving and cooling, add asparagine 50 mg/L, L-pyroglutamic acid 100 mg/L, IAA 0.1 mg/L, GA3 0.5 mg/L, ZR 1 mg/L, PG, 1 mg/L, ticarcillin 200 mg/L, and spectinomycin 40 mg/L. Pour into sterile 100 × 25 Petri plates.

Rooting Medium (RM1). Dissolve 4.4 g MS medium with vitamins, 20 g sucrose, 0.59 g MES in water to make a total volume of 1 L; pH to 5.7 with KOH. Divide the media into two 1 L bottles, 500 mL each. Add 3.5 g of Bacto-agar to each bottle. Keep the lid loose. After autoclaving and cooling, add asparagine 50 mg/L, L-pyroglutamic acid 100 mg/L, ticarcillin 200 mg/L, IBA 1 mg/L, and PG 1 mg/L. Pour into sterile 100 × 25 mm Petri plates.

Rooting Medium (RM2). Same as RM1, but without phloroglucinol.

Soil mixture. Combine Sunshine Mix #1 and Peat moss in a 3:1 mixture, respectively. Wet soil with fish based organic plant fertilizer (FOOP Organic Biosciences, Inc.). Watered plants weekly with this solution. If soil appears dry between FOOP watering, use distilled water to rehydrate. Do not over water.

Supplemental soil fertilizer. Fish-based organic plant fertilizer (FOOP Organic Biosciences, Inc.). Mix one capful of FOOP Garden One to one gallon of water, followed by addition of one capful of FOOP Garden Two. Plants are watered weekly with this solution. FOOP MIST is used weekly to gently wet the leaves of the soy in soil. Continue use until new plant growth is observed.

**Plant Materials and Growth Conditions**

Soybean (*Glycine max* (L.) Merr.) ‘MiniMax’ (PI 643148) seed used for the study was obtained directly from the Mathews lab [1], (ben.matthews@ars.usda.gov) on 1 January 2020. Can also be obtained by special request from the GRIN center through an ‘advanced’ search, or from the Thomson lab upon request. Plants are grown in the greenhouse with supplemental lighting for a 16/8 light–dark photoperiod at 24 °C in Sunshine Mix #1 for the production of seed used in these experiments. Three experiments plus a control were conducted using approximately 200 seeds per replicate.

**Construct genotype and expected phenotype**

*Agrobacterium* strain JGT44 was used for soybean transformation. This is a GA*A*NTRY [6] strain-modified version of EHA105 (unpublished). The JGT44 strain was engineered to provide positive selection for spectinomycin. The structure of the T-DNA is illustrated in Figure 3A. The Spec gene is expressed with the soybean ubiquitin3 promoter driving an aadA1 ORF codon optimized for soybean, and fused to a pea chloroplast transit peptide for spectinomycin selection during regeneration. The T-DNA contains two genes driven by the *Arabidopsis* 10 promoter, mCherry3 (mCH3) and BAG4 that are fused by the P2A peptide skipping element. mCherry3 is used for rapid visual selection of transgenic seed. BAG4 is an *Arabidopsis* derived Bcl-2-associated AthanoGene 4 gene that is an antiapoptotic agent used to increase rates of transformation. Renilla (Rluc) luciferase acts as a gene of interest and is expressed by the double enhanced cauliflower mosaic promoter db35S. Rluc is used to determine the level of gene expression based on the genomic location of T-DNA integration [24]. T-DNA sequence available upon request.

**Molecular analysis of the transformants**

To characterize transformants carrying the JGT44 construct, genomic DNA was extracted from the leaves of mCherry3-expressing plants with a DNA purification kit (Puregene-QIAGEN). Primers Gmubi600F60 (5′ GATTACTTCAGATCCGTTAAACGTAACCATAGATCAGG), Spec OPT 6300 R60 (5′ GGGATCTAAACGTCTACTTTCATCGAAAAAGGAATC), and Ubi3t 8550 R60 (5′ CCTATACTGTTCTCTGACAATGGTGGCTTC), BAG4 600 F60 (5′ GTCGGTAGCTTGAACCCTCCTCC), were used for confirmation, generating amplificons of 534 bp and 681 bp, respectively. Platinum SuperFi II polymerase (Fisher) and protocol were used with a 15 s extension time. An Eppendorf MasterCycler Gradient thermocycler was used for amplification. Amplicons were visualized after electrophoresis in a 0.8% TAE agarose gel (Figure 3C).

T-DNA copy number was determined by droplet digital PCR (ddPCR) using the lectin housekeeping gene [8] reference primers Gmlec1F (5′ CATGACTCCCCATGCATCAC), Gmlec1R (5′ CAGTAGCACCAGTACCAAGG), Gmlec1 Probe (5′ CTGAAGCAAAGCAATGGCTACTTCA—FAM), and specific primers Spec OPT P F56 (5′ ACGATCTGCTGGAGACTAGC), Spec OPT P R56 (5′ CACGGTATGATGTCGTCGTG), and SpecOPT Probe 65 (5′ ACCCAACACCTCTCTTGAGCGCA—HEX BHQ1) following Bio-RAD standard instructions for amplification. ddPCR was carried out using droplet generation oil with the microcapillary droplet generator cartridge, an Eppendorf MasterCycler Gradient thermocycler, Bio-RAD QX200 Droplet reader and analysed using QuantaSoft™ software (BioRad Laboratories, Inc. Hercules, CA, USA) (Figure 4A).

Bradford assay. Sample preparation: leaf tissue was flash frozen, ground in a mortar and pestle with liquid nitrogen, and stored at −80 °C. Extracted protein from 250 mg ground tissue was suspended in 1 mL Luciferase Extraction Buffer (Potassium phosphate 100 mM, EDTA 1 mM, glycerol 10% *v*/*v*, Triton-X 100 1% *v*/*v*, pH 7.8). BSA standards were prepared at 1500, 1000, 750, 500, 375, 250, 125, and 0 µg/mL by making serial dilutions of stock 2 mg/mL stock (Thermo Sci. Cat. 23209). We added 2 µL of BSA standard to 58 µL Bradford reagent (1:30) and incubated 10 min at room temperature. Then, 1.2 µL of each BSA standard was loaded into a DS-11+ Spectrophotometer (DeNovix, Inc. Wilmington, DE, USA), and a standard curve was generated using a built-in Bradford Assay Program. The same procedure as the BSA Standards was used to prepare and analyze soy protein samples. Samples were diluted beforehand with water as needed.

Rluc assay. The *Renilla* Luciferase Assay System was used (Promega, Madisin, WI, USA, Cat. No. E2810) following the manufacturer’s instructions, with the exception that the Luciferase Extraction Buffer (Potassium phosphate 100 mM, EDTA 1 mM, glycerol 10% *v*/*v*, Triton-X 100 1% *v*/*v*, pH 7.8) was used for tissue extraction instead of the buffer provided by the kit.

**Procedure**

The flow chart and timeline for *Agrobacterium*-mediated transformation of MiniMax mature seed are shown (Figure 1 and Figure 2). The ‘half seed’ transformation process is modified from the Wang lab [7]. The process takes 65 days from mature seeds to growth of T0 transgenic plants in soil and approximately 145 days for T1 seed harvest.

**Detailed transformation steps and time required**

Sterilization of soybean seeds with chlorine gas. Time: 1 day

i. Place mature soybean seeds in a sterile 100 × 20 mm Petri dish with about 150 seeds per dish. Write the seed name on Petri dish. Caution: Do not exceed approximately 200 seeds per Petri dish, as a larger number of seeds results in incomplete surface sterilization.
ii. In the fume hood, put the Petri dish with seeds in a 15 mL round bottom culture tube sterilization container and open the lid. Place a 250 mL beaker inside the sterilization container and add 100 mL of bleach containing 5.25% sodium hypochlorite.
iii. Add 4.5 mL 12N HCl to the beaker containing bleach and close the sterilization container immediately. The initial reaction will produce bubbles (chlorine gas).
iv. Allow the container to vent for 20 h to complete the reaction, eliminate the chlorine gas, and sterilize the seeds. Seeds should be surface-sterilized completely to avoid contamination.
v. Close the Petri dish in the sterilization container and transfer it to a laminar flow hood. Open the lid and allow to sit for 30 min or more. Close the Petri dish, seal with Parafilm, and store at 4 °C. Sterilized seeds can be stored at 4 °C in dry conditions for six months to a year.

Soaking seeds with sterile water. Time: 1 day

vi. In a laminar flow hood, add sterile water into 100 × 25 mm Petri dish containing 150–200 sterilized seeds until seeds are covered. Close and seal Petri dish with parafilm.
vii. Store at room temperature (22–25 °C) overnight (~20 h).

Half-seed explant preparation. Time: 3 h

viii. In a laminar flow hood, transfer the soaked seeds to a sterile Petri dish. Use a scalpel blade to make a longitudinal cut along the hilum to separate the cotyledons.
ix. Remove the seed coat and keep the embryo containing half-seed explants in the Petri dish. Cover with lid. Do not let explants dry out and turn brown; this will decrease the susceptibility of explants to *Agrobacterium* infection.

*Agrobacterium* preparation. Time: 3 days

x. Start *Agrobacterium* preparation 2 days before transformation. Streak *Agrobacterium*, in this case GA*A*NTRY strain JGT44 (Figure 3A), on an LB plate containing 200 mg/mL gentamicin. Incubate the plate for 1 d at 28 °C.
xi. Inoculate a single colony of *Agrobacterium* from the plate into 3 mL LB liquid medium containing 100 mg/mL gentamicin. Culture at 28 °C with shaking (250 rpm) for 36 h.
xii. Spin the agrobacterium culture at 3600 rpm for 10 min at room temperature. Remove the supernatant, resuspend the pellet in liquid IM medium, and adjust the OD (650 nm) to 0.6 with IM medium.
xiii. Gently shake the resulting infection medium at 60 rpm at room temperature for 2 h before use.

*Agrobacterium* infection and co-cultivation. Time: 6 d

xiv. Prepare co-culture medium plates. After autoclaving co-culture medium, add GA3 0.25 mg/L, BAP 1.67 mg/L, AS 40 mg/L, cysteine 400 mg/L and DTT 154.2 mg/L. Pour into 100 × 15 mm Petri plates. Overlay plate with sterile filter paper to reduce agrobacteria overgrowth after the medium is solidified.
xv. Transfer prepared half-seed explants into *Agrobacterium* resuspension liquid tube (around 100 explants per 50 mL tube), and cover with Parafilm. Make 2–4 holes in the parafilm and place under vacuum (200 mm Hg) (Stratagene, Vacuum Control Station) for 10 min. Remove from vacuum and incubate at room temperature for an additional 10 min.
xvi. After co-culture gently remove excess infection media with pipet. Transfer infected explants onto co-cultivation medium using sterile forceps, with the flat, adaxial side touching the filter paper, allowing roughly 6–9 explants per Petri dish plate. Do not transfer liquid with explants to co-cultivation plates, to avoid overgrowth of agrobacterium.
xvii. Wrap the plates with Parafilm and place at 24 °C under an 18/6 photoperiod for 5 days.

Shoot induction. Time: 28 days

xviii. After 5 days co-cultivation, place the explants on spectinomycin selection shoot induction medium (SI, 6–9 explants per plate). Explants should be oriented with the nodal end of the cotyledon embedded in the medium and the regeneration regions flush to the surface, with the flat side up at a 30–45° angle.

Note: If agrobacterium overgrowth is detected, briefly wash the half-seed explants in Shoot Induction Washing medium (SIW, 50 mL in a 100 × 25 sterile petri plate, room temperature) for 5 min. Up to 50 explants may be washed per 50 mL of SIW. Gently remove washing medium with sterile pipet. Repeat if SIW solution appears cloudy upon removal.

xix. Wrap each plate with microporous surgical tape and incubate at 24 °C, 18/6 photoperiod for 14 days. Explants should be transferred to fresh SI every 14 days.

Shoot elongation. Time: 14–28 days

xx. After 28 days on SI, transfer the explants to fresh shoot elongation medium (SE), seal each plate with microporous surgical tape, and incubate at 24 C, 18/6 photoperiod.
xxi. Transfer the tissue to fresh SE to maintain cultures. Seal each plate with microporous surgical tape and incubate at 24 °C, 18/6 photoperiod, under the same conditions described above. Repeat every 14 days.

Rooting of transgenic plants. Time: 14 days

xxii. When shoots surviving in spectinomycin selection medium reach at least 3 cm, excise them from the shoot pad. Using sterile forceps, transfer green shoots to 100 × 25 sterile Petri dish plates containing root initiation medium (RM) without selection. Wrap the plate with microporous surgical tape and incubate in growth chamber at 24 °C, 18/6 photoperiod, for 2–3 weeks. Shoots should continue developing in root initiation medium with spectinomycin selection. If plants appear weak, transfer to RM without transgenic selective agent (spectinomycin) to help shoot rooting in root step.
xxiii. Roots should start appearing after a week. When roots grow longer than 3 cm, and the shoots 4–5 cm, remove the plants from deep Petri dish with forceps. Wash briefly with running tap water to remove traces of Bacto-agar.
xxiv. Fill new pots with the ¾ Sunshine Mix 1: ¼ Peat Moss mix and moisten the soil with FOOP Garden solution. Make a hole in the soil (depth depending upon the root length) and place the regenerated shoot into the hole. Compact the soil around the shoot slightly.
xxv. Place containers with shoots into a growth chamber at 24 °C, with 18/6 photoperiod, covered with plastic dome for 1–2 weeks. Shoots should show some growth before being transferred to greenhouse conditions.
xxvi. FOOP is added to the soil upon transfer to solid medium and plants are misted weekly, according to the manufacturer’s instructions.

Grow transgenic plants in greenhouse and harvest T1 seeds. Time: 75 d

xxvii. After being in the growth chamber for 1–2 weeks, plants are transferred to big pots (6” H × 5” L × 8” D) and moved to the greenhouse with a 16/8 light–dark photoperiod at 24 °C.
xxviii. Harvest T1 seeds. After around 2 months, the mature T1 seeds can be harvested, Dry at 30 °C for 3–10 days.

Reproduce T1 seeds.

xxix. Separate mCherry3 positive transgenic T1 seeds under a Leica MZ16F florescence stereomicroscope at 1X magnification (Figure 3B). Plate the mCherry3 positive seeds in big pots containing the Sunshine Mix #1, with 2 plants per pot. Grow in a greenhouse with a 16/8 light–dark photoperiod at 24 °C.
xxx. After around 3 months, the mature T2 seeds can be harvested. Dry at 30 °C for 3–10 days.

## 5. Conclusions

We have demonstrated a modified ‘half seed’ regeneration protocol for transgenic soybean production utilizing the rapid generation MiniMax variety to obtain T1 seeds in approximately 145 days. The addition of PG to the regeneration protocol was key to obtaining high efficiency rooting, while use of an organic soil amendment containing mycorrhiza appeared necessary for plants to thrive in the greenhouse. This combination of protocol, genotype, and stimulants provides a means to accelerate research to test genes of interest for soybean trait improvement.

## Figures and Tables

**Table 1:** Regeneration and transformation efficiencies for MiniMax explants.

Replicate No.	Explant No.	Regeneration Shoots		Rooting Plants		mCherry3 Expressing Plants		Greenhouse Survival Plants		PCR Positive Plants	Transgenic Rate %
No.	No.	No.	Rate %	No.	Rate %	No.	Rate %	No.	Rate %	No.	Rate %
1	206	61	29.6	58	28.2	49	23.8	10	4.85	9	4.37
2	203	57	28.1	45	22.2	43	21.2	7	3.45	7	3.45
3	190	63	33.2	49	25.8	46	24.2	9	4.74	8	4.21
Total	599	181	30.2	152	25.4	138	23.0	26	4.34	24	4.01

**Table 2:** Media used for tissue culture and transformation of soybean MiniMax variety.

Medium	Composition (Basic)	Added after Autoclaving (Final Concentrations)
CCM	1/10 Gamborg B5 basal medium, 1 mL Gamborg vitamin (1 mg/mL), 30 g Sucrose, 3.9 g MES, 4.25 g Bacto-agar, pH to 5.4 with KOH.	GA3 0.25 mg/L, BAP 1.67 mg/L, AS 40 mg/L, L-Cysteine 400 mg/L, and DTT 154.2 mg/L
IM	1/10 Gamborg B5 basal medium, 1 mL Gamborg vitamin (1mg/mL), 30 g Sucrose, 3.9 g MES, pH to 5.4 with KOH.	GA3 0.25 mg/L, BAP 1.67 mg/L, AS 40 mg/L
SIW	Gamborg B5 medium with vitamins, 30 g Sucrose, 0.59 g MES, pH to 5.7 with KOH.	BAP 1.67 mg/L, ticarcillin 200 mg/L
SI	Gamborg B5 medium with vitamins, 30 g Sucrose, 0.59 g MES, 7 g Bacto-agar, pH to 5.7 with KOH.	BAP 1.67 mg/L, ticarcillin 200 mg/L, PG 1.0 mg/L
SE	MS medium with vitamins, 30 g Sucrose, 0.59 g MES, 7 g Bacto-agar, pH to 5.7 with KOH.	Asparagine 50 mg/L, L-Pyroglutamic Acid 100 mg/L, IAA 1 mg/L, GA3 0.5 mg/L, ZR 1 mg/L, Ticarcillin 200 mg/L, PG 1.0 mg/L
RM1	MS medium with vitamins, 20 g Sucrose, 0.59 g MES, 7 g Bacto-agar, pH to 5.6 with KOH.	AS 50 mg/L, L-Pyroglutamic Acid 100 mg/L, Ticarcillin 200 mg/L, IBA 1mg/L, PG 1.0 mg/L
RM2	MS medium with vitamins, 20 g Sucrose, 0.59 g MES, 7 g Bacto-agar, pH to 5.6 with KOH	AS 50 mg/L, L-Pyroglutamic Acid 100 mg/L, ticarcillin 200 mg/L, IBA 1mg/L

## Data Availability

Data are available upon request.

## Supplementary Materials

The following supporting information can be downloaded at: https://www.mdpi.com/article/10.3390/plants13071013/s1, Figure S1: Representative mCherry3 fluorescence images of leaves from wild type (WT) and JGT44 T0 transgenic lines; Figure S2: PCR analysis of genomic DNA of putative transgenic soybean plants using BAG4 gene and Spec gene primers. The length of PCR productions is 681 bp and 550 bp, respectively. M: DNA ladder; P: JGT44 gDNA; N: Wild type soybean gDNA; 1–24 are representative transformed JGT44 T0 soybean plant leaf Gdna; Table S1: Detailed preparation of media used for tissue culture and transformation of soybean MiniMax variety.
