# Supplementary material for: Agrobacterium-Mediated Transformation of the Dwarf Soybean MiniMax
Source: Plants (Basel). 2024 Apr 2;13(7):1013. doi: 10.3390/plants13071013 (PMC11013704; doi:10.3390/plants13071013)
Supplement: Supplementary file 1 [file plants-13-01013-s001.zip › plants-2872719-supplementary.pdf]

Agrobacterium-mediated Transformation of the dwarf Soybean MiniMax

mCherry3 fluorescence of JGT44 in T0 leaves of MiniMax

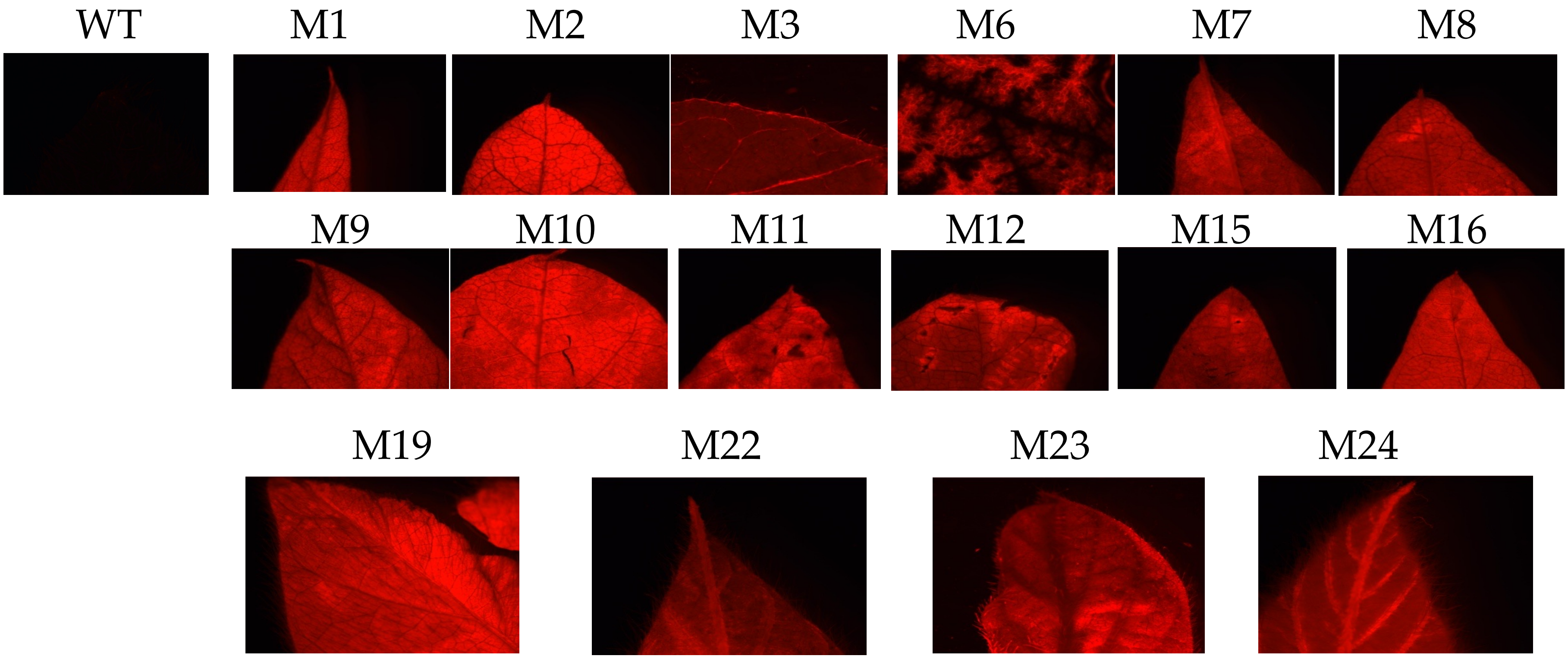

Supplemental Figure S1. Representative mCherry3 fluorescence images of leaves from wild type (WT) and JGT44 T0 transgenic lines.

Agrobacterium-mediated Transformation of the dwarf Soybean MiniMax

PCR analysis of JGT44 in T0 leaves of MiniMax

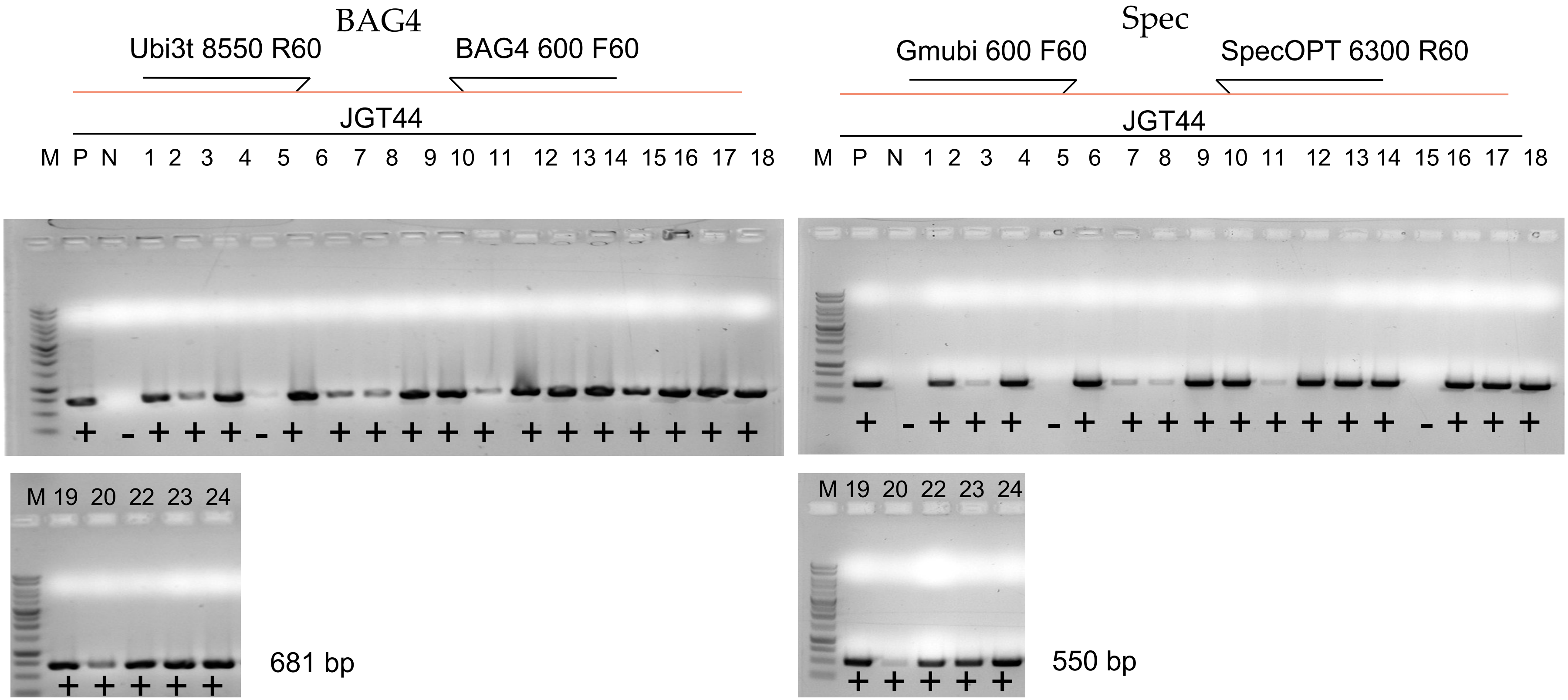

Supplemental Figure S2. PCR analysis of genomic DNA of putative transgenic soybean plants using BAG4 gene and Spec gene primers. The length of PCR productions is 681 bp and 550 bp, respectively. M: DNA ladder; P: JGT44 gDNA; N: Wild type soybean gDNA; 1 – 24 are representative transformed JGT44 T0 soybean plant leaf gDNA.

Agrobacterium-mediated Transformation of the dwarf Soybean MiniMax

6-Benzylaminopurine (BAP) (1 mg /mL; PhytoTech Labs). Dissolve 50 mg BAP in 50 mL distilled water (dH_2O) + 5 drops 37% HCl. After it has dissolved, filter sterilize with 0.2 µm Nylon sterile filter (Fisher)

Gibberellic acid (GA3) (1 mg/mL; PhytoTech Labs) Dissolve 10 mg GA3 in 1 ml of 100% ethanol, add 9 mL dH_2O to 10 mL. Filter sterilize.

Indole-3-butyric acid (IBA) (1 mg/ mL; PhytoTech Labs). Dissolve 10 mg IBA in 1 mL of 1 N NaOH. Add dH_2O to 10 mL. Filter sterilize.

Indole-3-acetic acid (IAA) (1 mg /mL; PhytoTech Labs). Dissolve 10 mg IAA in 5 mL of 100% ethanol. Add dH_2O to 10 mL. Filter sterilize.

Acetosyringone (AS) (40 mg/mL; PhytoTech Labs). Dissolve 400mg AS in DMSO 10 mL. Filter sterilize. Use freshly made, or divide into 1 ml aliquots and store at -20 C.

L-Cysteine (50 mg/ml; PhytoTech Labs). Dissolve 400 mg L-cysteine in 8 ml dH_2O. Filter sterilize.

Dithiothrietol (DTT) (154.2 mg/L; Sigma) Dissolve 1.542mg in 10 ml dH_2O. Filter sterilized.

Asparagine (50mg/ml; PhytoTech Labs). Dissolve 500mg asparagine in 5ml 1N NaOH, add dH_2O to 10 mL. Filter sterilize.

L-Pyroglutamic acid (100mg/mL; PhytoTech Labs). Dissolve 1g L-pyroglutamic acid in10 ml dH_2O. Filter sterilize.

After filter sterilization, keep all stock solutions at 4 C.

Supplemental Table S1. Detailed preparation of media used for tissue culture and transformation of soybean MiniMax variety

Agrobacterium-mediated Transformation of the dwarf Soybean MiniMax

trans-Zeatin-riboside (ZR) (1mg/mL; PhytoTech Labs). Dissolve 10 mg Zeatin-R in 1mL 1 N NaOH, add dH_2O to 10 mL. Filter sterilize.

Ticarcillin (200mg/mL; PhytoTech Labs). Dissolve 2g ticarcillin in 10 mL dH_2O, Filter sterilize.

Spectinomycin (100 mg/mL; PhytoTech Labs). Dissolve 1g spectinomycin in 10 mL dH_2O, Filter sterilize.

Myo-inositol (50 mg/mL; PhytoTech Labs). Dissolve 500 mg myo-inositol in 10 mL dH_2O, Filter sterilize.

Thiamine HCl (1 mg/mL; Sigma). Dissolve 10 mg thiamine HCl in 10 mL dH_2O, Filter sterilize.

Phloroglucinol (PG) (1 mg/mL; Sigma) Dissolve 10 mg PG in 1 mL 100% ethanol, add dH2O to 10 mL, Filter sterilize. PG is also resistant to autoclaving and is thus amenable to plant tissue culture as a non-sterile stock solution.

Gentamicin (100 mg/mL; PhytoTech Labs). Dissolve 1g gentamycin in 10 mL dH_2O, Filter sterilize.

Gamborg vitamin (1mg/mL; PhytoTech Labs).

After filter sterilization, keep all stock solutions at 4 C.

Supplemental Table S1 continue... Detailed preparation of media used for tissue culture and transformation of soybean MiniMax variety
